# Genomic Characterization of Urothelial Carcinoma and Sarcomatoid Carcinoma of the Upper Urinary Tract

**DOI:** 10.3390/cancers18142352

**Published:** 2026-07-21

**Authors:** Salvador Jaime-Casas, Nicholas J. Salgia, Miguel Zugman, Vitor Goes, Ali Moradi, Koral Shah, Rahul Winayak, Regina Barragan-Carrillo, Jadon Fann, George Zhang, Benjamin Mercier, Daniela V. Castro, Nazli Dizman, JoAnn Hsu, Alexander Chehrazi-Raffle, Tanya Dorff, Wesley Yip, Sumanta K. Pal, Abhishek Tripathi

**Affiliations:** 1 Department of Medical Oncology & Experimental Therapeutics, City of Hope Comprehensive Cancer Center, Duarte, CA 91010, USA; scasas@uw.edu (S.J.-C.);; 2 Department of Immunology, Roswell Park Comprehensive Cancer Center, Buffalo, NY 14263, USA; nicholas.salgia@roswellpark.org; 3 Instituto Nacional de Cancerologia (INCAN), Mexico City 14080, Mexico; 4 M.D. Anderson Cancer Center, Houston, TX 77030, USA; 5 Division of Urology and Urologic Oncology, Department of Surgery, City of Hope Comprehensive Cancer Center, Duarte, CA 91010, USA

**Keywords:** urothelial carcinoma, upper-tract, sarcomatoid, genomic landscape, targetable mutations

## Abstract

**Simple Summary:**

Sarcomatoid carcinoma of the upper urinary tract (SCUT) represents a rare and aggressive cancer subtype that remains poorly characterized. Using a large real-world genomic database, we compared the clinical and genomic features of 121 patients with SCUT and 1600 patients with upper tract urothelial carcinoma (UTUC). Patients with SCUT were younger at diagnosis, presented with more advanced disease, and demonstrated a distinct metastatic tropism, predominantly involving lung, bone, brain, and pleura. Compared to UTUC, SCUT tumors were enriched in *NF2*, *SETD2*, *PTEN*, *PBRM1*, and *BAP1*, while showing a notable depletion of *FGFR3*, *KMT2D*, and *ARID1A* alterations. Our finding establish SCUT as a distinct biologic entity and identify potentially actionable targets that could help inform clinical trial design.

**Abstract:**

Background: Sarcomatoid carcinoma of the upper urinary tract (SCUT) is a rare and aggressive malignancy. Due to its rarity, the molecular landscape and the prevalence of potentially targetable alterations are poorly characterized. We aimed to compare the clinical, pathological, and genomic profiles of SCUT and upper tract urothelial carcinoma (UTUC). Methods: We leveraged the Tempus Lens clinically annotated genomic dataset to extract clinicopathologic and somatic genomic alteration data from patients with UTUC and SCUT. Patients with any-stage disease who underwent either blood- or tissue-based next-generation sequencing were included. Baseline clinical and demographic characteristics were summarized using descriptive statistics. Comparisons between groups were performed using Wilcoxon rank-sum test for continuous variables and the Chi-square test/Fisher’s exact test for categorical variables. Mutational frequencies and pairwise comparisons were performed to assess significant differences between groups. Results: In total, 1721 patients were included, of which 1600 (93%) had UTUC and 121 (7%) had SCUT. Patients with SCUT were younger at diagnosis, 61 years (interquartile range (IQR) 54, 70), compared to UTUC, 71 years (IQR 64, 77) (*p* < 0.001), and were more likely to have node-positive disease at presentation (all *p* < 0.001). SCUT patients were more likely to show visceral metastases to the lung (44% vs. 21%), bone (31% vs. 17%), and brain (7% vs. 1%), compared to UTUC (all *p* < 0.05). Among SCUT patients, the most common genomic alterations were *TERT* (30%), *TP53* (29%), *NF2* (19%), *PTEN* (13%), *SETD2* (12%), *PBRM1* (12%), and *BAP1* (8%). Among UTUC patients, the most common were *TERT* (52%), *TP53* (52%), *KMT2D* (30%), *FGFR3* (25%), *ARID1A* (20%), and *KDM6A* (18%). Compared to UTUC, SCUT was significantly enriched with *NF2*, *SETD2*, *PBRM1*, *PTEN*, and *BAP1* (all *p* < 0.05). SCUT was depleted in *FGFR3* (0% vs. 25%) and *FGF4* (0% vs. 8%) mutations compared to UTUC (both *p* < 0.05). Targetable alterations were observed in SCUT, including *NF2*, *SETD2*, and *PTEN.* Conclusion: Compared with UTUC, SCUT exhibits a more aggressive clinical and genomic phenotype, characterized by enrichment in *NF2*, *SETD2*, *PBRM1*, and *PTEN.* These findings underscore the divergent molecular landscape of SCUT and highlight potentially targetable genomic alterations.

## 1. Introduction

Upper tract urothelial carcinoma (UTUC) originates within the pyelocaliceal system and ureter and represents a highly aggressive disease entity. While roughly 90% of cases are sporadic, approximately 10% of cases occur in the context of Lynch syndrome (hereditary non-polyposis colorectal cancer) [1,2]. UTUC exhibits a unique molecular pattern characterized by a higher frequency of *FGFR3* and *HRAS* mutations, which are hallmarks of aggressive disease biology [3,4,5]. While urothelial carcinoma is the most common tumor histology, variants showing divergent differentiation may manifest as mixed or pure populations. Among these, subtypes such as sarcomatoid, glandular, micropapillary, squamous, and lipid-rich variants have been described, each with distinct clinical course and varying degrees of sensitivity to systemic treatment [6,7,8]. Moreover, variant histology has been associated with adverse clinicopathological features, including advanced pathologic stage and grade, lymphovascular invasion on surgical specimens, and an increased risk of tumor recurrence [9,10].

Sarcomatoid carcinoma of the upper urinary tract (SCUT) is a rare and aggressive subtype composed of malignant epithelial and stromal components. However, due to the paucity of reported cases, most of the information pertaining to molecular alterations and potential therapies are often extrapolated from sarcomatoid differentiation of tumors originating in the bladder [11]. In this context, bladder tumors exhibiting sarcomatoid and urothelial components within the same tumor foci have been described as potentially sharing truncal somatic mutations, with additional alterations restricted to either component might suggest divergent evolution [12]. For example, sarcomatoid tumors express markers of active epithelial-to-mesenchymal transition (decrease in E-Cadherin and increase in TWIST1) as urothelial cells transition toward a sarcomatoid morphology. Similarly, urothelium-predominant tumors have higher expression of basal cytokeratins (KRT5, KRT6) compared to sarcomatoid tumors, reflecting their dedifferentiated nature [11,13].

A case series of eight patients demonstrated that patients with SCUT exhibited high rates of locally advanced disease after surgical resection (pT3/T4), evidence of nodal metastases, and a poor 2-year overall survival rate [14]. While some studies have attempted to elucidate potential tumor surface markers or genomic alterations that are therapeutically targetable, most evidence is extrapolated from patients with sarcomatoid carcinoma of the bladder [15].

Consequently, there is a significant gap in understanding the genomic landscape of SCUT and the potential actionable alterations that could inform therapeutic approaches. To address this, we sought to compare the clinical, pathological, and genomic profiles of SCUT and conventional UTUC.

## 2. Methods

### 2.1. Study Design and Data Source

We performed a retrospective analysis of the de-identified, clinically annotated, Tempus Lens data repository (queried September 2025), filtering for patients diagnosed with UTUC with or without sarcomatoid component. The Tempus Lens platform aggregates deidentified data from samples analyzed with the Tempus assay and enables real-time data visualization and analysis, and has been validated by various studies in this disease space [16,17,18]. Patients with a pure urothelial component were included in the UTUC group, and those with predominant sarcomatoid differentiation were assigned to the SCUT group. Inclusion criteria encompassed disease of any stage (T1–4, N0–2, M0–1) with available clinical and genomic data. Patients with incomplete clinicopathologic information and those without a histologic diagnosis were excluded. We extracted the somatic genomic with or without germline mutational profile. Somatic alterations were identified via next-generation sequencing of tissue (assays xT, xO, and xE) [19] derived from primary or metastatic sites, or via next-generation blood-based (assay xF) sequencing. The xT assay includes a 648-gene DNA sequencing panel for solid tumors, the xE assay incorporates a whole-exome 19,000+ gene DNA sequencing panel, the xF assay leverages a 105/523 ctDNA sequencing panel, and the xO assay combines a 1711-gene somatic and germline DNA sequencing panel with RNA sequencing [20]. For all eligible patients, we extracted clinicodemographic variables, including age at diagnosis, gender, race, ethnicity, and smoking status.

### 2.2. Statistical Analysis and Data Visualization

Clinical and demographic variables were summarized using descriptive statistics. Continuous variables are reported as medians and interquartile ranges (IQR), and categorical variables are reported as counts and percentages. Differences in the distribution of baseline characteristics between the cohorts were assessed with the Wilcoxon rank-sum test for continuous variables and the Chi-square test/Fisher’s exact test for categorical variables. For genomic alterations and metastatic site involvement, the Chi-square test/Fisher’s exact test was performed to compare the frequency of each alteration or site between groups. The decision between the Chi-square test/Fisher’s exact test was determined based on the number of patients in each group. Multiple hypothesis testing for genomic comparisons was controlled using the Benjamini–Hochberg false discovery rate (FDR) method. All tests were two-sided, and a *p*-value < 0.05 was considered statistically significant. All statistical analyses were performed in R Statistical Software (version 4.5.3).

Clinically significant mutational variants identified in the SCUT (N = 121) and UTUC (N = 1600) cohorts were visualized using custom oncoplot-style bar charts. For each cohort, variants were classified by alteration type, including deletions, amplifications, hemizygous loss, missense, frameshift, fusion, and splice site mutations. Categories for specific alterations were based on the standard nomenclature of variant annotations. Variants were then filtered to those occurring at a frequency of ≥1.5% within their respective cohort and ranked in descending order of prevalence. The percentage of affected cases within each cohort was displayed on the *x*-axis to facilitate proportional comparison across cohorts. This was developed using R Statistical Software (version 4.5.3).

## 3. Results

### 3.1. Patient Demographics and Clinicopathologic Features

A total of 1721 patients meeting the inclusion criteria were identified in Tempus Lens, of which 1600 (93%) had UTUC and 121 (7%) had SCUT. Overall, the majority of patients were male (57% UTUC and 66% SCUT) and White (50% UTUC and 46% SCUT). Patients with SCUT were more likely to be younger at diagnosis (median age 61 vs. 71 years, *p* < 0.001), and have clinical T2 (12% vs. 2%) and T3 (36% vs. 26%) disease at diagnosis compared to patients with UTUC (all *p* < 0.001). Patients with SCUT were more likely to have T4 (13% vs. 11%), N1 (13% vs. 9%), and M1 (19% vs. 13%) disease at diagnosis compared to patients with UTUC (both *p* < 0.05). Microsatellite Instability-High (MSI-H) status was more prevalent in patients with UTUC than in those with SCUT (3% vs. 1%), although the difference was not statistically significant (Table 1). Distinct patterns of metastatic spread were observed between groups. In line with a more aggressive clinical course, patients with SCUT were more likely to have visceral disease and metastasis to the lung (44% vs. 21%), pleura (6% vs. 1%), brain (7% vs. 1%), bone (31% vs. 17%), and pancreas (3% vs. <1%) were more common compared to patients with UTUC. Conversely, patients with UTUC predominantly exhibit lymphatic metastatic spread (37% vs. 12%), involving both regional and non-regional lymph nodes, and liver (15% vs. 11%) compared to patients with SCUT (*p* < 0.001).

### 3.2. Genomic Landscape

**Figure 1:**
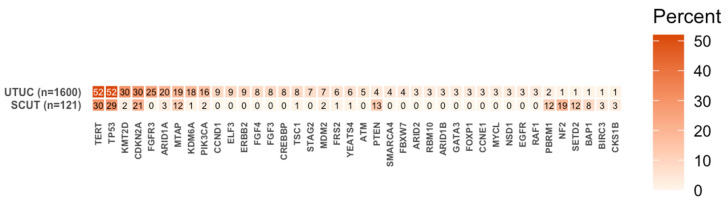
Prevalence of genomic alterations for patients with UTUC and SCUT.

Among SCUT patients, the most prevalent genomic alterations were *TERT* (30%), *TP53* (29%), *NF2* (19%), *PTEN* (13%), *SETD2* (12%), *PBRM1* (12%), *BAP1* (8%), and *ARID1A* (3%). Among UTUC patients, the most common genomic alterations were *TERT* (52%), *TP53* (52%), *KMT2D* (30%), *FGFR3* (25%), *ARID1A* (20%), and *KDM6A* (18%). Comparative analysis demonstrated that SCUT samples were significantly enriched for alterations in *NF2*, *SETD2*, *PBRM1*, *PTEN*, and *BAP1* (all *p* < 0.05). Compared to UTUC, SCUT samples showed significantly lower mutational rates in *FGFR3* and *FGF4* (both *p* < 0.05), with non-significant depletions also observed in *ERBB2*, *ATM*, *GATA3*, *FOXP1*, and *EGFR* (Table 2). Among SCUT samples, potentially targetable alterations included *NF2*, *SETD2*, and *PTEN* (Figure 1).

**Figure 2:**
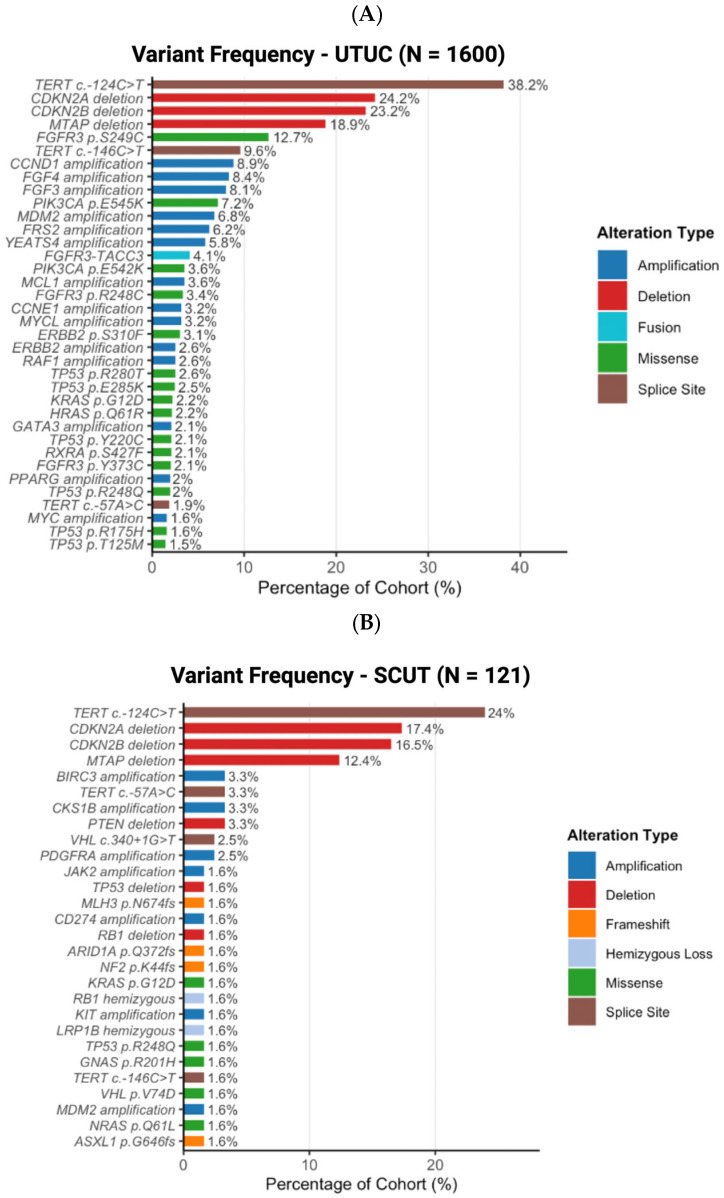
Clinically significant mutational variants frequency for UTUC (**A**) and SCUT (**B**) cohorts.

The frequency of clinically significant mutational variants across both cohorts are shown in Figure 2. In both the SCUT and UTUC cohorts, *TERT* c.-124C>T was the most frequently identified variant, occurring in 24.0% and 38.2% of cases, respectively. Deletions of *CDKN2A*, *CDKN2B*, and *MTAP* were the next most prevalent alterations in both groups, with higher frequencies observed in the UTUC group. Beyond these variants, the two cohorts demonstrated distinct mutational variant landscapes. For example, the UTUC cohort showed a high prevalence of *FGFR3* missense variants, including p.S249C (12.7%), p.R248C (3.4%), and p.Y373C (2.1%). In contrast, the SCUT cohort had a higher prevalence of splice-site variants, including *VHL* c.340+1G>T (2.5%).

## 4. Discussion

The present study compares the genomic landscape between patients with UTUC and SCUT. Our results show that patients with SCUT are significantly younger at diagnosis and present with a higher disease stage than patients with UTUC. Similarly, patients with SCUT show visceral metastatic tropism, with lung, pleura, bones, brain, and pancreas predominantly affected. Conversely, patients with UTUC predominantly exhibit lymphatic metastatic spread, involving both regional and non-regional lymph nodes. The distinct genomic mutational profiles of these disease types highlight their different biological characteristics and divergent differentiation.

Patients with SCUT show evidence of adverse clinicopathologic features, including a younger age, higher tumor stage, and nodal involvement at diagnosis. Sarcomatoid differentiation results from epithelial-to-mesenchymal transition (EMT), characterized by increased expression of tumor growth factor-β (TGF-β), epithelial growth factor (EGF), and insulin-like growth factor (IGF), along with reduced levels of epithelial cytokeratin markers. This transformation has been associated with advanced disease stages, aggressive clinical behavior, and poor oncologic outcomes in solid malignancies such as lung, breast, and colon cancers cancer [21,22,23]. In the context of bladder cancer, Liu and colleagues evaluated the prognostic significance of sarcomatoid differentiation and found that it was associated with worse 5-year cancer-specific survival and recurrence-free survival compared to patients with non-sarcomatoid carcinoma. Crucially, they observed a prognostic gradient based on the degree of histologic involvement, with patients with a low sarcomatoid component (1–50%) having a better prognosis than those with a high proportion (>50%) [24]. Similarly, Sirgiovanni et al. reported that within a cohort of 73 patients with high-grade urothelial carcinoma, the sarcomatoid subset (8%) universally exhibited advanced tumor stage, namely pT3 (67%) and pT4 (33%), and a high rate of regional lymph node involvement (67%) [15]. After a median follow-up of 33 months, all patients with sarcomatoid differentiation had local recurrence, irrespective of surgical pathology staging [15]. Similarly, Krabbe and colleagues compared outcomes and disease behavior between patients with UTUC and primary pelvicalyceal tumors against primary ureteral tumors. Interestingly, the number of altered markers and alteration status of markers were not significantly different between patients with primary pelvicalyceal versus ureteral tumors when stratified by tumor stage and nodal status, as well as no significant differences in survival outcomes between the two groups when stratified by the number of altered markers (p21, p27, p53, cyclin E, and Ki-67) [25]. Our findings mirror these results, as we identified higher disease stages at diagnosis and a metastatic profile with visceral tropism among patients with SCUT. This may indicate that sarcomatoid histology drives an inherently aggressive disease biology in urothelial carcinoma, regardless of the anatomic origin, and suggests that patients with SCUT could benefit from a more intense treatment pathway, due to the higher prevalence of advanced disease stage at diagnosis and visceral organ involvement.

Identifying specific clusters of signatures that correlate with tumor behavior clinically is of utmost importance for bridging genomic alterations with clinically actionable efforts. To this end, Fuji and colleagues performed a study of 199 UTUC samples to delineate the genetic landscape and classify tumors into five subtypes based on the mutational status of *TP53*, *MDM2*, *RAS*, and *FGFR3* [26]. For example, tumors harboring mutations in *TP53* and *CCND1* were more commonly associated with invasive UTUC, whereas alterations in *FGFR3*, *HRAS*, and *TERT* were more frequent in non-invasive tumors. This allowed them to describe specific clinical patterns associated with genomic alterations, yielding five unique clusters: hypermutated, *TP53/MDM2*-mutated, *RAS*-mutated, *FGFR3*-mutated, and triple-negative. Tumors pertaining to the *RAS*-mutated cohort frequently showed high-grade histology, squamous differentiation, and an overall aggressive phenotype. While extrapolating these clusters is unfeasible in our present cohort, these results underscore the importance of clustering genomic alterations based on pathways that could mechanistically explain clinical patterns. For example, patients with SCUT histology in our cohort harbored predominantly alterations in *TERT* and *TP53* and were largely devoid of *FGFR3* alterations, distinguishing them from the mutational pathways observed in UTUC.

Our results indicate a low incidence of MSI tumors in patients with UTUC and SCUT. MSI status is an important prognostic tumor market, as its presence has been associated with better outcomes and improved response to adjuvant chemotherapy and immunotherapy compared with tumors with stable microsatellite regions [27,28]. One notable example is the phase III KEYNOTE-045 trial of pembrolizumab versus paclitaxel, docetaxel, or vinflunine in recurrent advanced urothelial cancer. A total of 75 patients with upper tract disease were included, with results showing a significant clinical benefit of pembrolizumab over chemotherapy for both patients with upper tract and primary bladder carcinoma. While no histological or MSI substratification was performed for patients with upper tract carcinoma, it does provide an early signal to the potential role of immunotherapy in this patient population [29].

Patients with SCUT and UTUC reveal distinct metastatic patterns, with SCUT patients showing tropism for visceral involvement (lung, brain, bone, and pancreas), and UTUC patients showing lymphatic-predominant spread. This was recently analyzed by Drouaud and colleagues, who evaluated the impact of variant histology on metastatic sites at presentation in patients with bladder cancer. Their results found that distinct variant histology subgroups exhibited independent patterns of organotropism, with sarcomatoid histology showing a predominance of lung involvement as the primary metastatic site, followed by bone, nonregional lymph nodes, liver, and brain. Interestingly, this was in direct contrast to patients with other variant histologies, as patients with squamous cell and small cell variants showed tropism for the brain and liver, respectively, as their primary sites [30]. In a similar study of 398 patients with carcinoma of the upper urinary tract with variant histology, Rink et al. found that histological variants were associated with tumor multifocality, sessile tumor architecture, tumor necrosis, and lymphovascular invasion. Indeed, these adverse tumor-related characteristics are biologic elements that can predict a more aggressive disease course. Importantly, these are not mutually exclusive to any given variant, as tumors can harbor multiple histologic components [31]. To this end, a similar study by Holmäng et al., comparing patients with pure UTUC and squamous histology-predominant carcinoma of the upper urinary tract, found that patients with squamous differentiation had larger tumors at diagnosis and higher tumor stage, despite comparable rates of lymphovascular invasion [32]. Indeed, evidence supports the notion of an overlapping spectrum of adverse macro and microscopic pathologic features in variant histologies of the upper urinary tract. While the distinct mutational profile of SCUT may contribute to its metastatic patterns, further research is necessary to identify specific drivers of organotropism.

The distinct, non-overlapping mutational profiles between UTUC and SCUT highlight their divergent clonal origins and suggest a unique tumor biology that warrants distinct therapeutic considerations. In particular, we found that SCUT samples were depleted in potential targetable alterations, including *FGFR3*, *ERBB2*, *ATM*, *FOXP1*, and *EGFR*. The notable absence of *FGFR3* alterations in SCUT samples is particularly problematic, as this represents one of the primary therapeutic targets for drug design in upper tract disease. Due to the historical lack of clinical trials dedicated to SCUT, management has largely relied on extrapolating recommendations from conventional urothelial carcinoma. However, our analysis reveals that the non-overlapping profile might render this approach biologically unfounded. Future clinical trials must therefore prioritize designs that target specific genomic alterations enriched in SCUT samples, such as *NF2*, *SETD2*, and *PTEN.* While the therapeutic armamentarium targeting these alterations is currently limited and may be at different stages of the investigational pathway, we summarize a non-exhaustive list of clinical trials evaluating drugs that could potentially target these SCUT-enriched alterations in Table 3. While the present study does not establish causality between specific genomic alterations and clinical behavior, the distinct mutational profiles observed between SCUT and UTUC offer insight into the underlying biology of each entity. For example, *TERT* promoter mutations were the most frequently found variant across both cohorts. This was further described by Wang et al., who showed that *TERT* promoter mutations are more characteristic of UTUC and are associated with more aggressive disease [33]. Interestingly, the co-occurrence in the SCUT group further denotes a potential clonal origin between both entities. Conversely, FGFR3-related variants were found almost exclusively in the UTUC group, consistent with the published literature reporting that up to 75% of UTUC tumors exhibit this alteration. Clinically, Katims et al. also describe that *FGFR3*-mutated patients with UTUC exhibit a T-cell phenotype characterized by more exhausted Th17-like CD4 T cells, fewer regulatory T cells, and more CD8/cytotoxic cells in the naïve state, with a lower response to IL-1 and TNF, which may have clinical implications when exposed to immune system-stimulating drugs [34].

Our study is limited by its retrospective design. Since genomic profiling is not performed routinely across all centers, there may be selection bias in including patients treated at tertiary academic centers or those with more aggressive disease that warrant genomic analysis. Importantly, the dataset includes both tissue-based and blood-based sequencing assays, which can show sensitivity variations. The distribution of tissue-only and blood-only profiling across both cohorts was not available, which can introduce variability in detection rates and should be considered when interpreting inter-cohort genomic comparisons. It is also possible that among patients with SCUT, additional co-occurring histologies were present. Importantly, not all patients had clinical staging or metastatic data available, and comprehensive clinical variables like race, diagnostic and treatment details, and clinical course, were unavailable for patients, reflecting the inherent constraints of real-world genomic databases. Any given patient could have multiple sites of metastatic involvement, impacting the overall rates of metastases. Similarly, patients with predominantly sarcomatoid differentiation were included in the SCUT group, and no stratification based on percentage of sarcomatoid differentiation was performed. Finally, the absence of survival and treatment-related information precludes us from correlating genomic drivers with oncologic outcomes or longitudinal mutational profiles under treatment pressure. As such, all genomic findings should be interpreted as hypothesis-generating and require additional prospective validation before informing clinical decision-making. Additionally, effect sizes and confidence intervals could not be estimated, as alteration frequencies are reported at the cohort level.

## 5. Conclusions

To our knowledge, this is one of the largest genomic comparisons between SCUT and UTUC. Our findings reveal that SCUT is a distinct biological entity from UTUC, with more aggressive clinicopathologic behavior characterized by a younger age at diagnosis, higher tumor stage, and lymph node involvement. In addition, our results underscore the unique visceral metastatic organotropism of SCUT, which predominantly affects the lung, pleura, brain, bone, and pancreas. The depletion of *FGFR3*, *ERBB2*, *ATM*, *FOXP1*, and *EGFR* alterations in SCUT samples underscores the need to design trials tailored to individual mutational profiles and to consider alternative strategies, such as antigen mapping via immunohistochemistry, to leverage novel therapeutic options like immunotherapy and antibody-drug conjugates.

## Figures and Tables

**Table 1:** Baseline characteristics for patients with UTUC and SCUT.

Variable		UTUC (n = 1600)	SCUT (n = 121)	*p* Value
Age (median, IQR)				<0.001
		71 (64, 77)	61 (54, 70)	
Sex (No. %)				0.06
	Female	686 (43%)	41 (34%)	
	Male	914 (57%)	80 (66%)	
	Missing	0 (0%)	0 (0%)	
Race (No. %)				0.19
	White	800 (50%)	56 (46%)	
	Other Race	62 (4%)	4 (3%)	
	Black or African American	57 (4%)	9 (7%)	
	Asian	52 (3%)	3 (3%)	
	Missing	629 (39%)	49 (41%)	
Ethnicity (No. %)				0.07
	Not Hispanic or Latino	650 (41%)	48 (40%)	
	Hispanic or Latino	52 (3%)	8 (6%)	
	Missing	898 (56%)	65 (54%)	
ECOG Score (No. %)				0.91
	1	627 (39%)	46 (38%)	
	0	543 (34%)	35 (29%)	
	2	256 (16%)	16 (13%)	
	3	85 (5%)	4 (3%)	
	4	13 (1%)	0 (0%)	
	Missing	76 (5%)	20 (17%)	
Smoking Status (No. %)				0.35
	Never smoker	245 (16%)	26 (21%)	
	Ex-smoker	70 (4%)	9 (8%)	
	Current smoker	62 (4%)	3 (2%)	
	Missing	1223 (76%)	83 (69%)	
MSI (No. %)				0.36
	Stable	1272 (80%)	88 (73%)	
	High	50 (3%)	1 (1%)	
	Missing	278 (17%)	32 (26%)	
T Stage (No. %)				<0.001
	Ta/Tis	104 (7%)	0 (0%)	
	T1	143 (9%)	1 (1%)	
	T2	186 (12%)	2 (2%)	
	T3	590 (36%)	32 (26%)	
	T4	175 (11%)	16 (13%)	
	Missing	402 (25%)	70 (58%)	
N Stage (No. %)				<0.001
	N0	423 (26%)	16 (13%)	
	N1	144 (9%)	16 (13%)	
	N2	97 (6%)	0 (0%)	
	N3	6 (<1%)	0 (0%)	
	NX	289 (18%)	29 (24%)	
	Missing	641 (40%)	60 (50%)	
M Stage (No. %)				<0.001
	M0	365 (23%)	11 (9%)	
	M1	203 (13%)	23 (19%)	
	Mx	109 (7%)	2 (2%)	
	Missing	923 (57%)	85 (70%)	
Metastatic site (No.%)				<0.001
	Lymph nodes	595 (37%)	14 (12%)	
	Lung	334 (21%)	53 (44%)	
	Bone	264 (17%)	38 (31%)	
	Liver	247 (15%)	13 (11%)	
	Soft tissue	58 (4%)	14 (12%)	
	Other soft tissue	246 (15%)	37 (30%)	

ECOG, Eastern Cooperative Oncology Group; IQR, interquartile range; MSI, microsatellite instability; SCUT, sarcomatoid carcinoma of the upper tract; UTUC, upper tract urothelial carcinoma.

**Table 2:** Prevalence of the most common genomic alterations in patients with UTUC and SCUT.

Gene	UTUC (n = 1600)	SCUT (n = 121)	*p* Value
*TERT*	827 (52%)	36 (30%)	<0.001
*NF2*	18 (1%)	23 (19%)	<0.001
*KMT2D*	484 (30%)	2 (2%)	<0.001
*FGFR3*	396 (25%)	0 (0%)	<0.001
*SETD2*	15 (1%)	14 (12%)	<0.001
*TP53*	832 (52%)	35 (29%)	<0.001
*KDM6A*	287 (18%)	1 (1%)	<0.001
*PBRM1*	40 (2%)	15 (12%)	<0.001
*ARID1A*	323 (20%)	4 (3%)	<0.001
*PTEN*	69 (4%)	16 (13%)	<0.001
*BAP1*	23 (1%)	10 (8%)	<0.001
*PIK3CA*	261 (16%)	3 (2%)	<0.001
*CCND1*	143 (9%)	0 (0%)	0.01
*ELF3*	140 (9%)	0 (0%)	0.01
*ERBB2*	137 (9%)	0 (0%)	0.01
*FGF4*	134 (8%)	0 (0%)	0.01
*FGF3*	129 (8%)	0 (0%)	0.01
*CREBBP*	121 (8%)	0 (0%)	0.02
*STAG2*	113 (7%)	0 (0%)	0.03
*TSC1*	136 (8%)	1 (1%)	0.03
*SMARCA4*	66 (4%)	0 (0%)	0.07
*ATM*	82 (5%)	0 (0%)	0.11
*FBXW7*	64 (4%)	0 (0%)	0.11
*BIRC3*	12 (1%)	4 (3%)	0.11
*FRS2*	100 (6%)	1 (1%)	0.12
*ARID2*	54 (3%)	0 (0%)	0.14
*YEATS4*	93 (6%)	1 (1%)	0.16
*CKS1B*	15 (1%)	4 (3%)	0.16
*MDM2*	109 (7%)	2 (2%)	0.16
*RBM10*	49 (3%)	0 (0%)	0.16
*ARID1B*	48 (3%)	0 (0%)	0.16
*GATA3*	48 (3%)	0 (0%)	0.16
*FOXP1*	50 (3%)	0 (0%)	0.16
*CCNE1*	51 (3%)	0 (0%)	0.16
*MYCL*	51 (3%)	0 (0%)	0.16
*CDKN2A*	481 (30%)	26 (21%)	0.20
*NSD1*	42 (3%)	0 (0%)	0.23
*MTAP*	312 (19%)	15 (12%)	0.23
*EGFR*	47 (3%)	0 (0%)	0.23
*RAF1*	47 (3%)	0 (0%)	0.23

**Table 3:** Non-exhaustive list of clinical trials exploring therapeutic targets for actionable mutations in SCUT.

Mutational Pathway	Drug	Clinical Trial/NCT	Population	Design
NF2-related pathways	Crizotinib	LCI-GU-URO-CRI-001/NCT02612194	Metastatic urothelial carcinoma of the bladder, upper or lower urinary tracts	Phase II
	Everolimus	RAD001/NCT00805129	Advanced urothelial carcinoma	Phase II
		NCT01184326	Previously treated advanced urothelial carcinoma	Phase I
		HCRN GU10-147/NCT01215136	Cisplatin ineligible advanced urothelial carcinoma	Phase II
SETD2-related pathways	Talozaparib	TALASUR/NCT04678362	Platinum-sensitive metastatic or locally advanced urothelial carcinoma	Phase II
	Decitabine	NCT00030615	Advanced solid tumors	Phase I
PTEN-related pathways	X31005	NCT03745911	Metastatic urothelial carcinoma	Phase II

## Data Availability

The data that support the findings of this study are available upon reasonable request from the corresponding author.
